# SOCS2 regulation of growth hormone signaling requires a canonical interaction with phosphotyrosine

**DOI:** 10.1042/BSR20221683

**Published:** 2022-12-09

**Authors:** Kunlun Li, Lizeth G. Meza Guzman, Lachlan Whitehead, Evelyn Leong, Andrew Kueh, Warren S. Alexander, Nadia J. Kershaw, Jeffrey J. Babon, Karen Doggett, Sandra E. Nicholson

**Affiliations:** 1 The Walter and Eliza Hall Institute of Medical Research, Parkville, Australia; 2 Department of Medical Biology, University of Melbourne, Parkville, Australia

**Keywords:** growth hormone, phosphotyrosine, SH2 domain, SOCS2

## Abstract

Suppressor of cytokine signaling (SOCS) 2 is the critical negative regulator of growth hormone (GH) and prolactin signaling. Mice lacking SOCS2 display gigantism with increased body weight and length, and an enhanced response to GH treatment. Here, we characterized mice carrying a germ-line R96C mutation within the SOCS2-SH2 domain, which disrupts the ability of SOCS2 to interact with tyrosine-phosphorylated targets. *Socs2^R96C/R96C^* mice displayed a similar increase in growth as previously observed in SOCS2 null (*Socs2^−/−^*) mice, with a proportional increase in body and organ weight, and bone length. Embryonic fibroblasts isolated from *Socs2^R96C/R96C^* and *Socs2^−/−^* mice also showed a comparable increase in phosphorylation of STAT5 following GH stimulation, indicating the critical role of phosphotyrosine binding in SOCS2 function.

## Introduction

Growth hormone (GH) is secreted by the anterior pituitary gland into the circulation, binding to a homodimeric GH receptor (GHR) on the surface of cells throughout the body to regulate growth, metabolism, and the immune response. In 1987, the GHR became the first cytokine/hematopoietin receptor superfamily member to be cloned, initiating a molecular investigation into GH signaling [1]. Despite this, it took many years to fully delineate mechanisms of ligand recruitment and signal propagation [2,3]. GHR engagement promotes a conformational change in the homodimeric receptor complex, activating the receptor-associated Janus kinase 2 (JAK2) tyrosine kinases [2,3]. Activated JAK2 in turn phosphorylates tyrosine residues within the GHR cytoplasmic domain, recruiting Src homology 2 (SH2)-containing proteins such as the signal transducers and activators of transcription (STAT) proteins to the receptor complex [4]. Once STAT5b homodimers are recruited to the receptor complex, they are phosphorylated by JAK2, triggering a conformational dimer change, translocation to the nucleus, and the transcription of genes involved in growth and development, such as insulin-like growth factor 1, *Igf1* [5–7]. Excessive GH production results in acromegaly in humans and is associated with severe pathological consequences [5–7], underscoring the need for cellular mechanisms that limit the GH response.

The suppressor of cytokine signaling (SOCS) protein family, consisting of CIS and SOCS1-7, functions to limit cytokine signaling, often in a negative feedback loop [8]. The important role of SOCS2 as a negative regulator of GH signaling was revealed by SOCS2-deficient mice, which displayed a 30–40% increase in growth compared with wild-type (WT) mice [9]. The gigantism was rescued by crossing SOCS2-deficient mice with *Ghrhr*^*lit/lit*^ (*little*) mice that lacked pituitary-derived GH secretion, with both *Ghrhr*^*lit/lit*^ and *Ghrhr*^*lit/lit*^ *Socs2^−/−^* mice exhibiting comparable growth retardation [10]. Similarly, SOCS2-null mice on a STAT5b-deficient background displayed a similar growth rate to WT mice, confirming SOCS2-deficient gigantism results from aberrant activation of GH signaling [11]. Notably, the increased weight of *Socs2^−/−^* mice was not due to excess fat gain, but resulted from a proportional increase in organ size, including muscle and bone length. Consistent with this, SOCS2-deficient mice have longer femur, tibia, radius, and humerus bones [9], associated with increased bone mass, but no change in bone mineral density [12]. However, others have reported reduced bone mineral density [13], with Dobie et al., reporting a decrease in cortical bone mineral density [14].

SOCS2 has a short 32-residue N-terminal region and two major functional domains: a central SH2 domain and a SOCS box. The SOCS2-SH2 domain is a substrate recognition module that binds to phosphorylated tyrosine residues 487 and 595 within the GHR cytoplastic domain, with SOCS2-binding proposed to inhibit cascade propagation via blocking access to other signaling intermediates [10]. The SOCS2-SH2 domain has also been reported to directly interact with JAK2 [15]. The SOCS box provides binding sites for the adaptors Elongins B and C, and the Cullin 5 scaffold, which in turn, recruit RING-box protein (Rbx)2 to form an E3 ubiquitin ligase complex that ubiquitinates substrates bound to the SH2 domain [16]. There is an underlying assumption that SOCS2 ubiquitinates the GHR complex directing it to proteasomal degradation.

The SOCS2-SH2 domain structure consists of three β-strands flanked by two α-helices, and an additional ‘SOCS-specific’ α-helix termed the extended SH2 subdomain (ESS) [17]. Interaction with phosphorylated targets occurs via a positively charged pocket (P0) that binds phosphorylated tyrosine (pTyr) and a hydrophobic patch that accommodates the third residue (+3) distal from the pTyr. Recently, we identified a noncanonical exosite on the SOCS2-SH2 domain that when occupied, enhances SH2 affinity for tyrosine phosphorylated targets [18]. Multiple SOCS2 residues, including Arg96, co-ordinate binding to the pTyr residue [19]. A naturally occurring mutation of Arg96 to Cys in ovine SOCS2 is associated with inflammatory mastitis, and increased body size and milk production [20], with mutation of Arg96 disrupting SH2 binding to phosphopeptides [18,20]. The characterization of the R96C mutation in sheep was the first study to investigate the contribution of SH2:pTyr binding to SOCS2 function *in vivo* [20]. However, there are no comparative studies of SOCS2-deficient sheep to address whether SOCS2 function is completely reliant on its SH2 interaction with phosphotyrosine.

Here, we generated and characterized mice bearing the SOCS2-R96C mutation (*Socs2^R96C^*). Homozygous *Socs2^R96C/R96C^* mice displayed a 25–35% increase in weight compared with WT mice during puberty, and a similar increase in body weight to SOCS2-deficient mice. The gigantism resulted from a collective increase in weight of most visceral organs, associated with increased body and bone length. GH signaling was enhanced in *Socs2^R96C/R96C^* fibroblasts, indicating that the augmented growth during development was a consequence of a dysregulated GH response. The *Socs2^R96C/R96C^* mice displayed a similar phenotype to *Socs2^−/−^* mice, highlighting a critical role for SOCS2-SH2 recognition of phosphotyrosine in canonical SOCS2 function.

## Results

### Mutation of R96 in SOCS2 does not disrupt SH2 domain integrity

**Figure 1:**
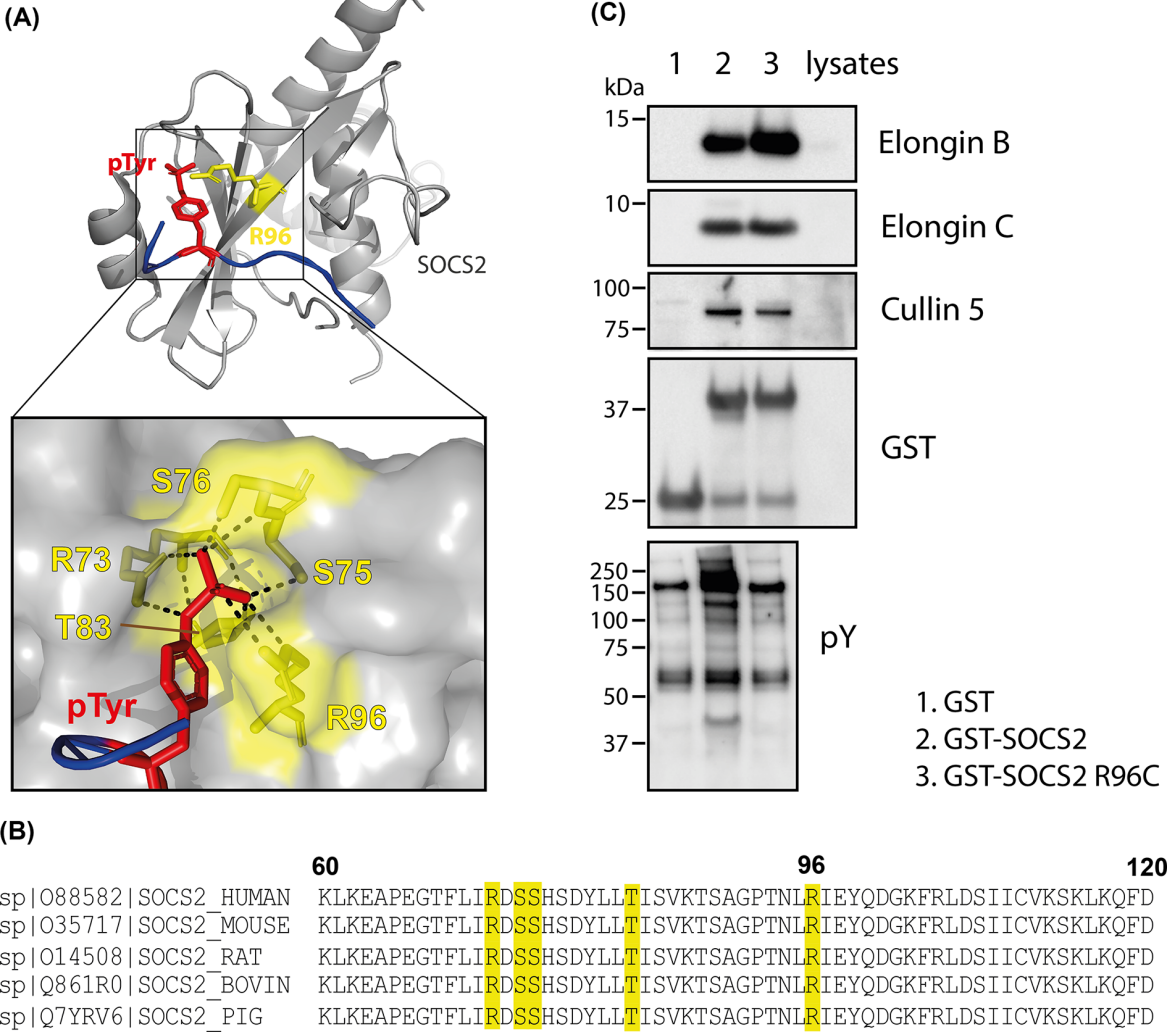
Arg96 within SOCS2 contributes to pTyr binding but not to SOCS box recruitment of the E3 ubiquitin ligase complex (**A**) Crystal structure of SOCS2 bound to a phosphopeptide derived from Y426 in the erythropoietin receptor. Inset is a surface representation of the SOCS2-SH2 domain P0 pocket, showing the hydrogen-bond interactions (dashed lines) with phosphotyrosine (pY426 EPO-R peptide: red stick; Arg96: yellow stick). (Kung et al. [19]; PDB 6I4X). (**B**) Alignment of SOCS2 sequences from different species. Multiple sequences were obtained from UniProt database and aligned using UniProt alignment tool. Residues interacting with phosphotyrosine are highlighted in yellow. (**C**) Affinity enrichment of recombinant GST-SOCS2/BC and GST-SOCS2-R96C/BC protein complexes was performed from 293T cell lysates. GST-SOCS2 enrichment of interacting proteins was analyzed by immunoblotting with antibodies to Elongin B, Elongin C, Cullin 5, and phosphotyrosine.

SOCS2-SH2 binding to pTyr occurs through a cluster of residues consisting of Arg73, Ser75, Ser76, Thr83, and Arg96 in the SOCS2-SH2 domain [19] (Figure 1A), with all five residues conserved across different species (Figure 1B). Although Arg73 corresponds to the invariant arginine residue found in all SH2 domains [21–23], mutation of Arg73 to Lys in SOCS2 resulted in reduced affinity for a phosphopeptide derived from GHR Y595 (pY595), but not loss of binding (Supplementary Figure 1).

We had previously shown that mutation of Arg96 to Cys in the SOCS2-SH2 domain abrogated binding to a phosphopeptide derived from GHR Tyr595, without impacting on peptide binding to the SH2 exosite, confirming domain integrity [18]. To verify that the R96C mutation did not impact SOCS box function, GST-SOCS2 and GST-SOCS2-R96C, were purified in a trimeric complex with Elongins B and C, and used to affinity precipitate Cullin 5 from cell lysates. Elongins B and C were present at comparable levels in both GST-SOCS2 and GST-SOCS2-R96C complexes. GST-SOCS2-R96C efficiently enriched Cullin 5 to the same extent as GST-SOCS2, evidence that mutation of R96C within the SOCS2-SH2 domain did not disrupt the SOCS box E3 complex (Figure 1C). GST-SOCS2-R96C failed to enrich tyrosine-phosphorylated proteins, confirming that pTyr binding was disrupted by the R96C mutation (Figure 1C).

### Gigantism in mice bearing a homozygous SOCS2 R96C mutation

**Figure 2:**
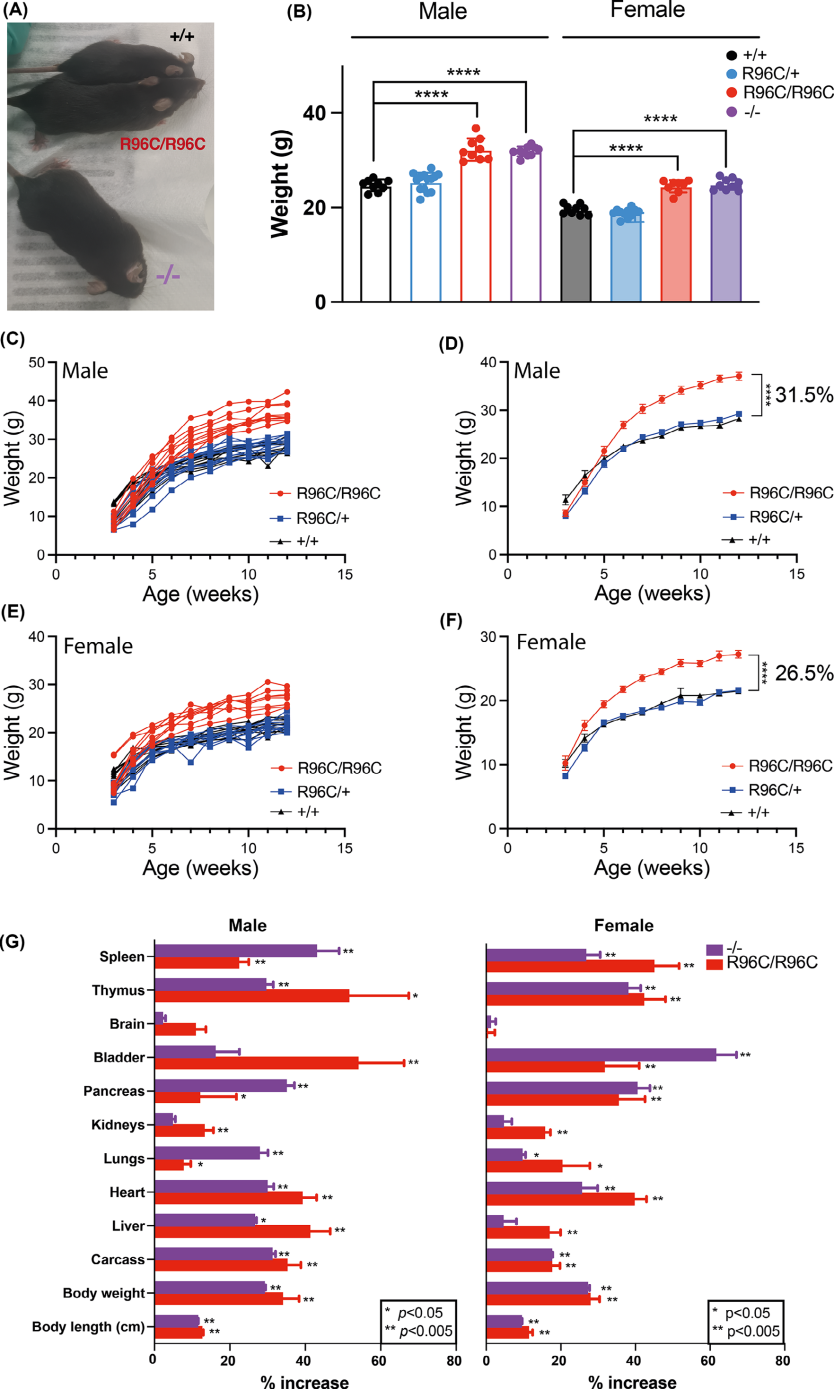
Homozygous *Socs2^R96C/R96C^* mice display enhanced growth consistent with dysregulated GH signaling during puberty (**A**) 13-week-old WT (+/+), *Socs2^R96C/R96C^*, and *Socs2^−/−^* male mice. (**B**) Body weights of 8-week-old WT (+/+), *Socs2^R96C/R96C^*, and *Socs2^−/−^* mice. Each dot represents an individual mouse. (**C**) Growth curves from male littermates: *Socs2^R96C/R96C^* (*n*=9), *Socs2^R96C/+^* (*n*=14), *Socs2^+/+^* (*n*=9). (**D**) Data from panel C as mean ± S.E.M. (**E**) Growth curves from female littermates: *Socs2^R96C/R96C^* (*n*=8), *Socs2^R96C/+^* (*n*=11), *Socs2^+/+^* (*n*=9). (**F**) Data from panel E as mean ± S.E.M. Mice were weighed weekly from 3 weeks of age. (**D,F**) Data were analyzed using a one-way ANOVA. ****P<0.0001. (**G**) Body length and carcass and organ weights were measured for 13-week-old male *Socs2^R96C/R96C^* (*n*=7), *Socs2^−/−^* (*n*=3), and *Socs2^+/+^* (*n*=7) mice, and female *Socs2^R96C/R96C^* (*n*=8), *Socs2^−/−^* (*n*=3), and *Socs2^+/+^* (*n*=6) mice. Data are shown as mean ± S.E.M. and expressed as a % of WT averages. Data were analyzed using an unpaired student *t*-test. *P<0.05, **P<0.005, ***P<0.0005, ****P<0.00005.

To further investigate the impact of mutating Arg96 to Cys *in vivo*, we used CRISPR/Cas9 gene editing to generate a C57BL/6 mouse strain bearing the R96C mutation. Correct gene targeting was validated by next-generation sequencing (NGS), with routine genotyping performed by PCR (Supplementary Figure 2). Homozygous *Socs2^R96C/R96C^* mice were viable and born at Mendelian frequencies. *Socs2^R96C/R96C^* mice were indistinguishable to *Socs2^R96C/+^* and WT mice before weaning at 3 weeks of age, but subsequently developed at a faster rate (Figure 2). Analysis of immune cell populations from *Socs2^−/−^* and *Socs2^R96C/R96C^* mice showed increased numbers of splenic natural killer (NK) cells (Supplementary Figure 3), consistent with previous observations [15]. By 6 weeks of age, both male and female *Socs2^R96C/R96C^* mice weighed significantly more than *Socs2^R96C/+^* or WT mice. The enhanced growth of *Socs2^R96C/R96C^* mice was evident throughout puberty, with growth plateauing at week 8, and maintained in adult mice (Figure 2C–F). At 8 weeks of age, body weights of *Socs2^R96C/R96C^* mice were comparable to *Socs2^−/−^* mice, with both significantly heavier than WT mice (30.7% and 29.1% in male, 25.4% and 27.5% in female, respectively), suggesting that the weight difference in *Socs2^R96C/R96C^* mice phenocopied *Socs2^−/−^* mice (Figure 2B). At 12 weeks of age, *Socs2^R96C/R96C^* male and female mice were visually different from WT mice (Figure 2A and not shown), with *Socs2^R96C/R96C^* male mice attaining a 31.5% weight increase over WT males (*Socs2^R96C/R96C^*: 37.1 ± 2.42 g, WT: 28.2 ± 1.39 g) (Figure 2C,D). *Socs2^R96C/R96C^* females typically attained the weight of WT male mice and displayed a 26.5% weight increase over WT females at 12 weeks of age (*Socs2^R96C/R96C^*: 27.2 ± 1.53 g, WT: 21.5 ± 0.98 g) (Figure 2E,F). Male and female *Socs2^R96C/+^* mice exhibited similar development and adult body weights to WT mice (Figure 2C–F). The increased size of *Socs2^R96C/R96C^* mice was reflected in a proportional increase in the weight of individual organs, with the majority of organs (excluding brain) showing increased weight compared with organs from WT mice. Pancreas and testes in males did not show a statistical difference. Carcass weight and body length were also increased in *Socs2^R96C/R96C^* mice. *Socs2^R96C/R96C^* mice displayed a similar increase to *Socs2^−/−^* mice, in organ weight, carcass weight, and body length (Figure 2G).

**Figure 3:**
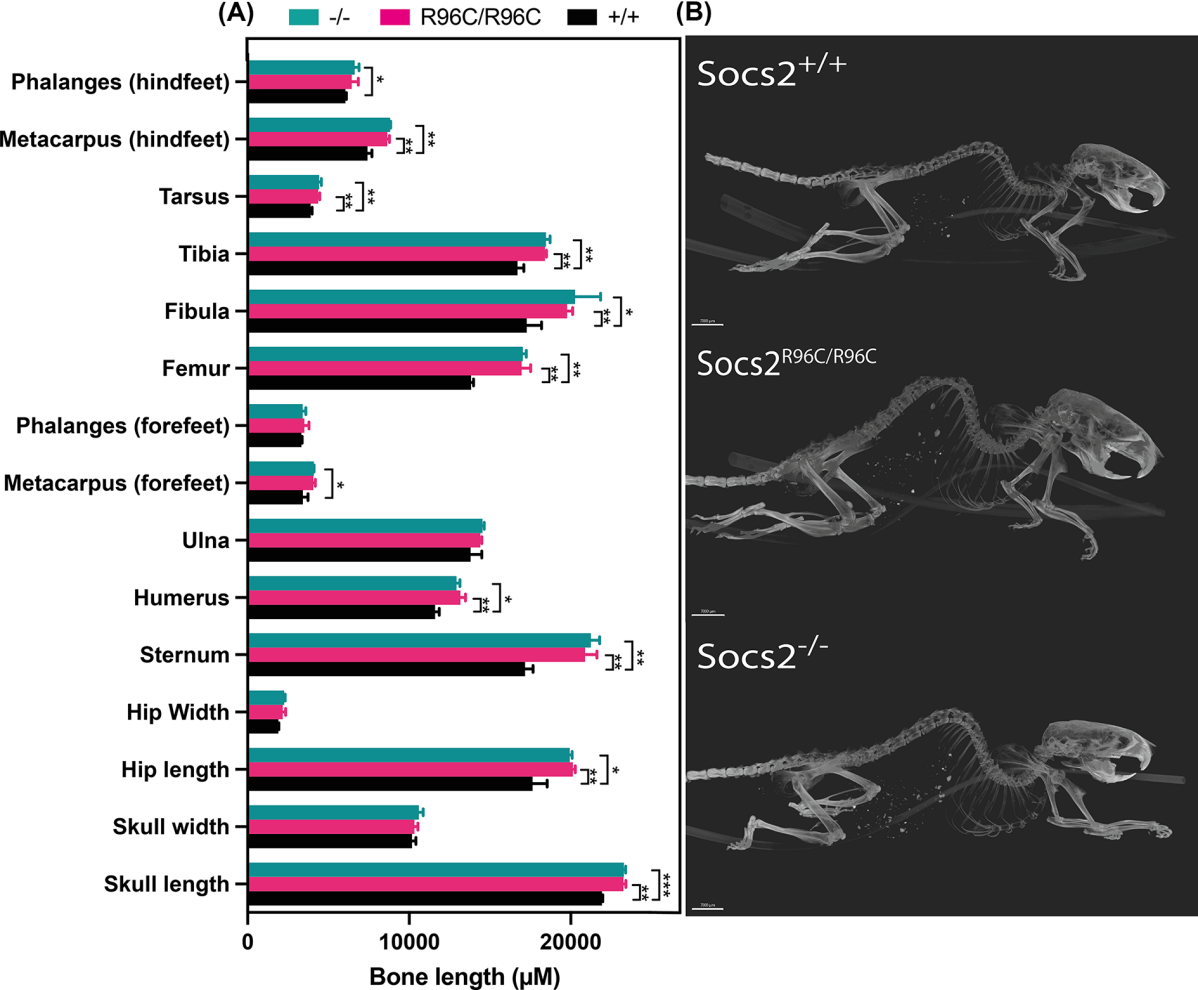
Homozygous Socs2-R96C mice display enhanced bone growth 14-week-old *Socs2^R96C/R96C^*, *Socs2^−/−^*, and *Socs2^+/+^* male mice were euthanized and whole animals analyzed by micro-CT. Skeleton X-ray imaging in 3D was collected using a Bruker Skyscan 1276 micro-CT. The X-ray projection images were reconstructed into 3D volumes using Bruker’s NRecon software, skeletons were visualized, and individual bone lengths measured in Imaris 8.7. (**A**) Bone lengths are shown as mean ± S.D. (*n*=3) and analyzed using an unpaired student *t*-test. *P<0.05, **P<0.005. (**B**) Example micro-CT images.

Micro-computed tomography (CT) X-ray imaging of skeletons from 13-week-old male WT, *Socs2^−/−^* and *Socs2^R96C/R96C^* mice revealed an enlarged skeleton and increased bone length of skull, hip, sternum, humerus, femur, fibula, tibia, tarsus, and metacarpus (hindfeet) in *Socs2^−/−^* and *Socs2^R96C/R96C^* mice. *Socs2^R96C/R96C^* mice showed comparable bone lengths to *Socs2^−/−^* mice (Figure 3). Histology of skin from 10-week-old *Socs2^R96/R96C^* male mice revealed dermal thickening and increased collagen production, comparable to that observed in *Socs2^−/−^* mice (Supplementary Figure 4), and consistent with previously reported skin pathology in *Socs2^−/−^* mice [9].

### Loss of SOCS2-SH2:pTyr-binding results in enhanced GH signaling

**Figure 4:**
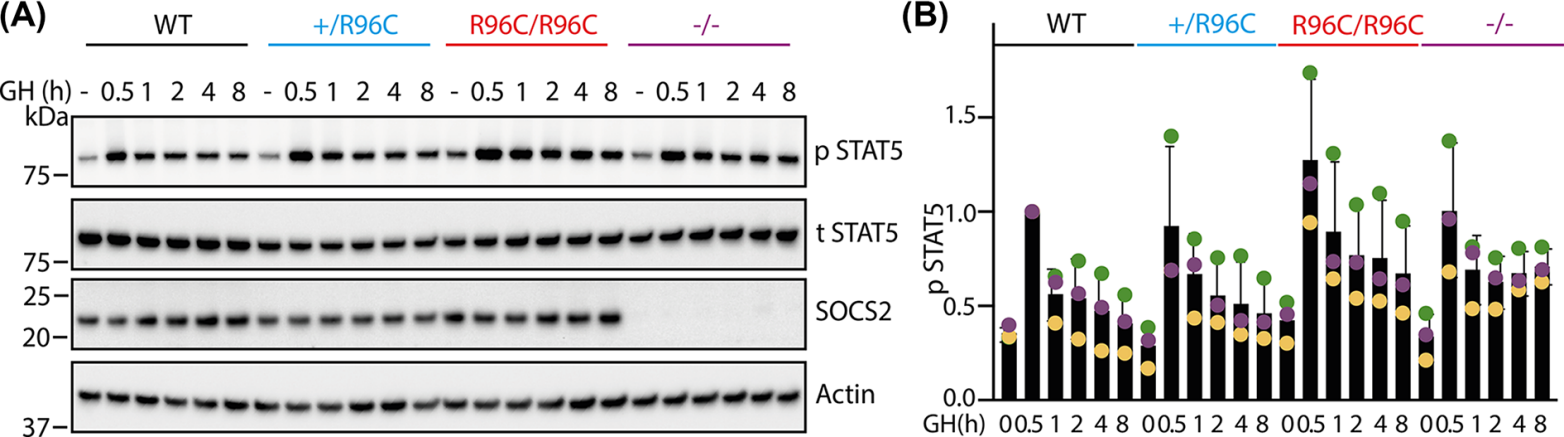
*Socs2^R96C/R96C^* and *Socs2^−/−^* MEFs display prolonged GH signalling *Socs2^+/+^*, *Socs2^R96C/+^*, *Socs2^R96C/R96C^*, and *Socs2^−/−^* MEFs were treated with and without (-) 50 ng/mL GH, lysed and analyzed by immunoblotting with antibodies to the indicated proteins. **(A)** Immunoblotting results of GH induction. p: phosphorylated, t: total. Representative of three independent experiments. **(B)** Quantification of phosphorylated STAT5 by densitometry (from Figure 4A and Supplementary Figure 5). Dots with same color indicate results from the same experiment.

To further investigate the impact of the SOCS2-R96C mutation on GH signaling, mouse embryonic fibroblasts (MEFs) were generated from *Socs2^+/+^, Socs2^R96C/+^*, *Socs2^R96C/R96C^*, and *Socs2^−/−^* day-13 embryos. GH stimulation resulted in robust tyrosine phosphorylation of STAT5 (pSTAT5; Figure 4 and Supplementary Figure 5), which peaked at 0.5 h GH treatment in MEFs of all genotypes. The intensity of the pSTAT5 response was comparable in *Socs2^R96C/R96C^* and *Socs2^−/−^* MEFs, and consistently increased at 1–8 h GH treatment, relative to *Socs2^R96C/+^* and *Socs2^+/+^* MEFs. *Socs2^R96C/+^* MEFs displayed an intermediate level of pSTAT5 compared with WT and *Socs2^R96C/R96C^* MEFs. Total STAT5 levels were comparable in all genotypes. SOCS2 was present at baseline, reduced in *Socs2^R96C/+^* MEFs and expressed at comparable levels in WT and *Socs2^R96C/R96C^* MEFs. There was a modest up-regulation of SOCS2 at 8 h. These data indicate that loss of phosphotyrosine binding is sufficient to abrogate SOCS2 regulation of GH signaling.

## Discussion

Five residues (Arg73, Ser75, Ser76, Thr83, and Arg96) within the SOCS2-SH2 domain form hydrogen bonds that lock the phosphorylated tyrosine residue in place [19]. As shown here, and in other studies [18,20], mutation of Arg96 to Cys results in complete loss of SOCS2-SH2 binding to phosphotyrosine and a corresponding loss of SOCS2 function. Mutation of Arg96 to Leu retained some weak binding, while R96Q completely abrogated binding to phosphotyrosine [19]. In cells, mutation of Arg73 to Lys has only a modest impact on SOCS2 regulation of GH signaling, unless combined with D74E and S75C.

We had previously shown that the SOCS2-R96C mutation did not impact on F3 peptide binding to the SOCS2 exosite [18]. In the present study, we confirmed the R96C mutation did not alter the ability of SOCS2 to recruit the SOCS box-associated-E3 ubiquitin ligase complex. Taken together, this confirmed the R96C mutation did not compromise domain integrity, enabling us to interrogate the role of SOCS2-SH2:pTyr binding in mice. *Socs2^R96C/R96C^* mice displayed increased growth during puberty, attaining a ∼25% and ∼30% increase in weight (females and males) compared with heterozygous littermates. The enhancement in body size and weight was comparable to SOCS2 null mice. In addition, primary embryonic fibroblasts derived from *Socs2^R96C/R96C^* mice displayed prolonged GH-induced signaling compared with WT cells, consistent with loss of a negative regulator (SOCS2), and further evidence that loss of phosphotyrosine binding was sufficient to fully disrupt SOCS2 function.

The structure of SOCS2 in complex with the GHR pY595 peptide indicated that SOCS2 has the capacity to not only engage phosphopeptide in the conventional manner but can also interact with a second GHR pY595 peptide orientated in an antiparallel direction. This model provides a potential mechanism for SOCS2 interaction with both subunits in the dimerized GHR [19]. In addition to pTyr binding, the SOCS2-SH2 domain can also bind to a nonphosphorylated peptide via the exosite, providing an alternative site for SH2 interaction with protein targets [18]. These two noncanonical interaction sites within the SOCS2-SH2 domain suggest that there may be additional protein interactions and regulatory mechanisms that impact on SOCS2 function. While our results confirm the critical role of SOCS2 in regulating GH signaling, the genetic relationship to mastitis in sheep (a bacterial infection of the udder) [20], indicates SOCS2 also has an important role in regulating the immune response to bacterial infection, most likely related to its up-regulation by LPS and IFNγ, and inhibition of NFkB signaling [24]. This suggests that SOCS2 regulates proteins independently of GHR-signaling complexes and this may occur either through classic SH2:pTyr interactions or via noncanonical SH2 interactions.

*SOCS2* has been associated with reduced growth in children and diverse disease pathologies. Given that mouse and human *SOCS2* share 94% sequence similarity, they are likely to have a conserved role as a critical negative regulator of GH signaling [18]. This is illustrated by correlation of a *SOCS2* polymorphism with a positive response to GH therapy during puberty, and increased adult height of children with GH deficiency and Turner syndrome after long-term rhGH treatment [25]. Thus, inhibition or reduction in SOCS2 has the potential to enhance the efficiency of long-term rhGH therapy. *SOCS2* mRNA levels were decreased in osteoarthritis patients [26], and a polymorphism in the *SOCS2* gene correlated with susceptibility to type 2 diabetes in a Japanese population [27–31]. Reduced SOCS2 protein expression is associated with a poorer outcome in diverse human cancers, such as colorectal cancer [31], breast carcinoma [32], and hepatocellular carcinoma [33], suggesting SOCS2 may have some utility as a prognostic biomarker.

SOCS2 is also an important regulator in the immune system [34]. DC-mediated T cell priming and adaptive antitumoral immunity is enhanced in SOCS2-deficient mice, resulting in reduced tumor burden [35]. Loss of SOCS2 in CD4^+^ T cells results in increased T helper (Th) 2 cell differentiation and allergic inflammation [27], while *Socs2^−/−^* CD4^+^ cells also show reduced Treg differentiation *in vivo* and *in vitro* under TGFβ stimulation [31]. *Socs2^−/−^* mice display increased numbers of NK cells (consistent with our observations) and up-regulated JAK2 and STAT5 activation in response to IL-15 [15].

The canonical role of SOCS2 in regulating GH signaling cannot account for the diverse role of SOCS2 in immunity and inflammatory diseases. The *Socs2^R96C/R96C^* mice provide an accessible model to investigate candidate pTyr targets for the SOCS2-SH2 domain *in vivo*, which will enable us to dissect the different pathways regulated by SOCS2, as well as the identification of novel SOCS2 targets.

## Methods

### Protein purification

pGEX4T constructs for expression of GST-human SOCS2 (residues 32-198), SOCS2-R73K (residues 32-198), and SOCS2-R96C (residues 32-198), and pACYCDuet constructs for expression of human Elongin B (residues 1-118) and Elongin C (residues 17-112), have been described previously [18]. The GST-SOCS2/BC trimeric complex was produced by co-expression in *E. coli* and purified by affinity chromatography as described previously [18]. The R73K mutation was introduced using the QuikChange Lightning Site-Directed Mutagenesis Kit (Agilent Technologies). The primer for mutagenesis (AATGCGAGCTATCTTTAATCAAGAAAGTTCCTTCTGGTGCC) was from Integrated DNA Technologies (Singapore).

### GST affinity precipitation

Human embryonic kidney cells (HEK293T) were pretreated with 10 μM MG132 for 4 h and sodium pervanadate for the final 30 min [36]. 2 × 10^7^ cells were lysed in 1 mL NP-40 lysis buffer (1% v/v NP-40, 50 mM HEPES, pH 7.4, 150 mM NaCl, 1 mM EDTA, 10% glycerol, 1 mM PMSF, 1 mM Na_3_VO_4_, 1 mM NaF) and protease inhibitors (Complete Cocktail tablets, Roche). Cell lysates were clarified by centrifugation at 13000 *g* for 15 min at 4°C. Supernatant was precleared with glutathione-sepharose resin (GE Healthcare). A total of 5 μg GST or 10 μg GST-SOCS2 protein was then added to cell lysates and incubated for 2.5 h at 4°C. A total of 30 μL of 50% glutathione sepharose resin (GE Healthcare) was then added and incubated for 60 min at 4°C. Glutathione sepharose resins were then washed with 3 × 1 mL of NP-40 lysis buffer and boiled with 20 μL SDS sample buffer.

### Surface plasmon resonance

The affinity of SOCS2 and SOCS2-R73K protein for a phosphopeptide derived from the GHR receptor was measured by competitive surface plasmon resonance (SPR), as described previously [18]. Briefly, biotin-GSGS-GHR pY595 peptide was immobilized to a streptavidin-coated SA chip and 100 nM of SOCS2 proteins preincubated with titrations of GHR pY595 competitor peptide (10, 3.3, 1.1, 0.3, 0.1 μM) was flowed over the chip. Peptides were purchased from Genscript.

### Mice

Mice carrying a germline mutation of Arg96 to Cys in SOCS2 (Socs2^R96C^) were generated on a C57BL/6J background by the MAGEC laboratory at the Walter & Eliza Hall Institute, as previously described [37]. A total of 20 ng/μL Cas9 mRNA, 10 ng/μL single-guide RNA (CAGCTGGACCGACTAACCTG), and 40 ng/μL oligo donor template (TCGCATTCAGACTACCTACTAACTATATCCGTTAAGACGTCAGCTGGACCGACTAAtCTatGtATTGAGTACCAAGATGGGAAATTCAGATTGGATTCTATCATATGTGTCAAGTCCAAGCTT) were injected into fertilized one-cell stage embryos. Additional silent changes were incorporated into the donor template to enable genotyping by PCR. Founder mice were analyzed by NGS to confirm the correct sequence change. Mice carrying the mutation were backcrossed to C57BL/6J mice for three generations to eliminate potential off-target events, and NGS repeated. Heterozygous *Socs2*^*R96C/+*^ mice were intercrossed to generate a homozygous *Socs2*^*R96C/R96C*^ line. Mice were routinely genotyped using genomic DNA extracted from ear biopsies with the Direct PCR Lysis tail reagent (Viagen) and 5 mg/mL proteinase K (Worthington), according to the manufacturer’s instructions. Sequencing primers are shown in Supplementary Table 1. *Socs2^−/−^* mice have been described previously [9], and both *Socs2^−/−^* and WT C57BL/6 mice were bred and housed in the same room as *Socs2^R96C^* mice. Experiments were performed at the Walter and Eliza Hall Institute (WEHI) in accordance with the NHMRC Australian code for the care and use of animals for scientific purposes. All experiments were approved by the WEHI Animal Ethics Committee (AEC 2021.002). Animals were euthanised using CO_2_ inhalation.

### micro-CT

14-week-old *Socs2^R96C/R96C^* and WT mice (*n*=3) were euthanized, and skeletal bones analyzed in intact carcasses by skeleton X-ray imaging in 3D using a Bruker Skyscan 1276 micro-CT. X-ray images were reconstructed into 3D volumes using Bruker’s NRecon software, skeletons were visualized, and length of individual bones measured in Imaris 8.7.

### Skin histology

Dorsal skin sections were taken from 10-week-old male *Socs2^R96C/R96C^, Socs2^−/−^*, and WT mice (*n*=3), fixed with 10% buffered formalin, embedded in paraffin, and stained with Van Giessen stain using standard techniques. Images were captured using the Panoramic Scan II (3D HISTECH Ltd.).

### Immunophenotyping

Spleens, axial, and inguinal lymph nodes, and cardiac bleeds were collected from 8- to 12-week-old mice. Single-cell suspensions from spleens and lymph nodes were acquired by mashing organs through a 70 µM cell strainer with FACS buffer (phosphate-buffered saline (PBS) + 2% FCS). Cardiac bleeds were collected in K3-EDTA tubes, and whole blood separated by density gradient, underlaying Histopaque®-1077 (Sigma-Aldrich) and centrifuging at room temperature for 15 min at 400× *g* with low acceleration and brake. The opaque interface was collected and washed twice with FACS buffer. Spleen and lymph node samples were resuspended in 5 mL of red-cell removal buffer (156 mM NH_4_Cl, 11.9 mM NaHCO_3_, and 0.097 mM EDTA) for 5 min at room temperature and washed twice with PBS.

Single-cell suspensions were resuspended in FACS buffer and 20 µL removed to determine absolute cell numbers, using 123count eBeads (Invitrogen™; 10000 beads/tube) and propidium iodine (1 µg/mL). The remaining cells were incubated with Zombie UV (1:1000 in PBS) for 15 min at room temperature, washed twice with FACS buffer, FC blocked (FcR Blocking Reagent, mouse, Miltenyi; 1:100 in FACS buffer) for 10 min at 4°C in the dark, and stained for 30 min at 4°C with the relevant antibody cocktail (Table 1). Cells were washed twice with PBS and fixed with 100 µL BD Cytofix™ Fixation Buffer for 30 min at 4°C. Fluorescent labeling and fixation was performed in the dark. Finally, cells were washed twice with PBS and analyzed using a BD FACSymphony™ cell analyser. Data analysis was performed with FlowJo software v10.

**Table 1:** Antibodies used in flow cytometric analysis

Antigen	Fluorophore	Dilution	Clone	Company	Catalog number
NK1.1	FITC	1:100	PK136	BD^*^	553164
TCR	percp-cy5.5	1:400	H57-597	BD	560657
CD3e	percp-cy5.5	1:400	145-2C11	BD	551163
CD4	BV421	1:1000	RM4-5	Biolegend	100546
CD8a	BV650	1:800	-	BD	563234
CD49b	APC	1:200	DX5	BD	560628
CD19	AF700	1:200	1D3	BD	557958
CD45	APC-CY7	1:200	30-F11	BD	557569
NKp46	PE/CY7	1:100	29a1.4	eBioscience	25-3351-82

* BD Biosciences.

### Isolation of primary MEFs and GH stimulation

Male and female *Socs2^R96C/+^* mice were set-up in timed matings, and e13 embryos harvested to generate *Socs2^+/+^*, *Socs2^R96C/+^*, and *Socs2^R96C/R96C^* MEFs. *Socs2^−/−^* MEFs were generated from *Socs2^−/−^* matings. Heads and fetal livers were removed, and embryos mechanically disaggregated and digested using trypsin, before culturing in tissue culture plates coated in 0.1% gelatin/PBS. MEFs were cultured in DMEM (Thermo) supplemented with 100 U/mL penicillin, 0.1 ng/mL streptomycin, and 10% FBS (Thermo) at 37°C in a humidified incubator with 10% CO_2_. MEFs at passage 5 were treated with 50 ng/mL of human GH for 0.5–8 h, lysed in NP-40 lysis buffer, and analyzed by immunoblotting. Human GH was kindly provided by Dr Andrew Brooks.

### Immunoblotting

Cell lysates were separated by sodium dodecyl sulfate-polyacrylamide gel electrophoresis (SDS-PAGE) and electrophoretically transferred to nitrocellulose membranes (Amersham). Membranes were blocked overnight in 5% w/v BSA and incubated with primary antibody for 2 h. Anti-GST-HRP (RPN1236, 1:10000) was from Sigma-Aldrich. Anti-phosphotyrosine antibody (4G10) was obtained from Millipore. Anti-Elongin C antibody (610761, 1:3000) was from BD Biosciences. Anti-p44/42 (9102S, 1:2000), anti-STAT5 (94205; 1:3000), and anti-phospho-STAT5 (9359; 1:2000) were purchased from Cell Signaling. Anti-actin-HRP antibody (C4) was obtained from Santa Cruz (sc-47778 HRP; 1:1000). Anti-Cullin 5 (ab184177), anti-Elongin B antibody (ab154854, 1:2000), and anti-SOCS2 antibody (ab109245, 1:1000) were purchased from Abcam. Antibody binding was visualized with peroxidase-conjugated sheep antirabbit immunoglobulin (Southern Biotech; 4010-05; 1:15000), or sheep antimouse immunoglobulin (GE Healthcare; NA931-1ML; 1:10000) and the enhanced chemiluminescence (ECL) system (Amersham or Millipore).

### Statistical analysis

Statistical analyses were performed with Prism 9 software. All statistical parameters including the exact value of *n*, the statistical test, error bars, and significance are reported in associated figure legends.

## Supplementary Material

Supplementary Figures S1-S5 and Table S1

## Data Availability

All supporting data are included within the main article and Supplementary file.

## Open Access

Open access for the present article was enabled by the participation of University of Melbourne in an all-inclusive *Read & Publish* agreement with Portland Press and the Biochemical Society under a transformative agreement with CAUL.

## Abbreviations

ANOVA: analysis of variance
CT: computed tomography
ECL: enhanced chemiluminescence
ESS: extended SH2 subdomain
FCS: fetal calf serum
GH: growth hormone
GHR: homodimeric GH receptor
HEK: human embryonic kidney cell
Igf1: insulin-like growth factor 1
JAK2: Janus kinase 2
LPS: lipopolysaccharide
MEF: mouse embryonic fibroblast
NGS: next-generation sequencing
NK: natural killer
P0: positively charged pocket
PBS: phosphate buffered saline
pTyr: phosphorylated tyrosine
S.D.: standard deviation
S.E.M.: standard error of the mean
SH2: Src homology 2
SOCS: suppressor of cytokine signaling
SPR: surface plasmon resonance
STAT: signal transducers and activators of transcription
Th: T helper
WT: wild-type
