# Supplementary material for: SOCS2 regulation of growth hormone signaling requires a canonical interaction with phosphotyrosine
Source: Biosci Rep. 2022 Dec 9;42(12):BSR20221683. doi: 10.1042/BSR20221683 (PMC9742514; doi:10.1042/BSR20221683)
Supplement: Supplementary Figures S1-S5 and Table S1 [file BSR-2022-1683_supp.pdf]

Supplementary - SOCS2 regulation of growth hormone signaling requires a canonical interaction with phosphotyrosine

Kunlun Li[1,2], Lizeth G. Meza Guzman[1,2], Lachlan Whitehead[1,2], Evelyn Leong[1], Andrew Kueh, Warren S. Alexander[1,2], Nadia J. Kershaw[1,2], Jeffrey J. Babon[1,2], Karen Doggett[1,2]†, Sandra E. Nicholson[1,2]†

[1]The Walter and Eliza Hall Institute of Medical Research, Parkville, Australia;
[2]Department of Medical Biology, University of Melbourne, Parkville, Australia.

† These authors jointly supervised this work and are corresponding authors.

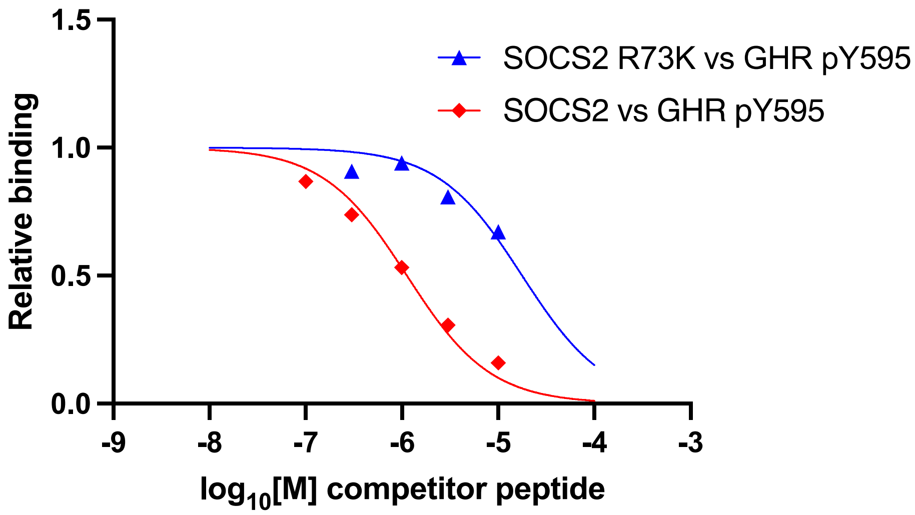

Supplementary Figure 1. Mutation of Arg73 to Lys in the SOCS2-SH2 domain results in reduced binding to phosphopeptide. A competitive surface plasmon resonance (SPR) assay was used to assess the impact of the R73K mutation. SOCS2 bound to a phosphopeptide derived from GHR pY595 with an IC_{50} 1.1 ± 0.05 µM. The SOCS2-R73K mutation reduced binding to GHR pY595, IC_{50} 26.7 ± 1.0 µM. IC_{50} values are mean ± S.D., derived from three independent experiments.

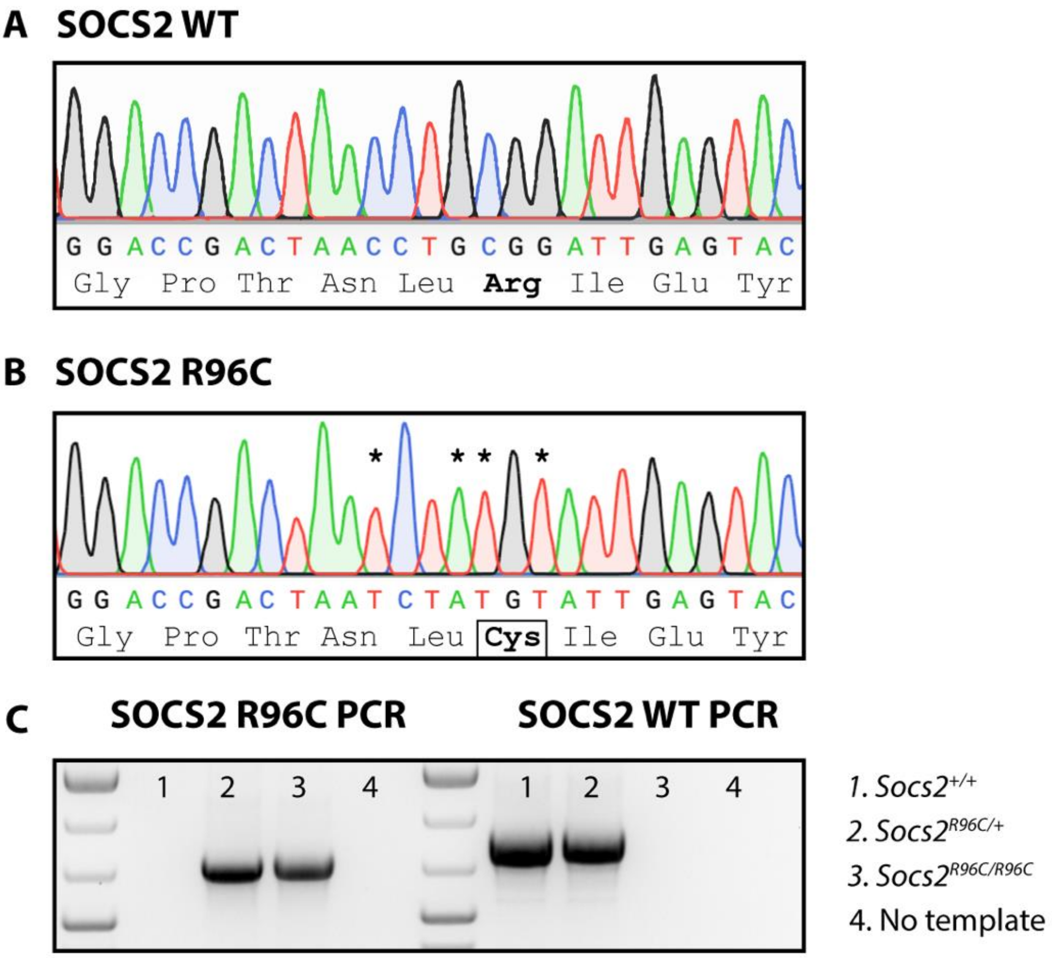

Supplementary Figure 2. Genotyping confirmation of the correct sequence change in the *$Socs2^{R96C}$* mouse. Sanger sequencing chromatograms confirming **(A)** the WT *Socs2* sequence and **(B)** the mutated *$Socs2^{R96C}$* sequence (homozygous mutant allele). *Highlights the CRISPR targeted base residue changes, two of which are synonymous and were introduced to enable primer specificity for standard gDNA genotyping. **(C)** Example 2% agarose gel confirming genotyping specificity by PCR. Primer sequences are available in **Supplementary Table 1**.

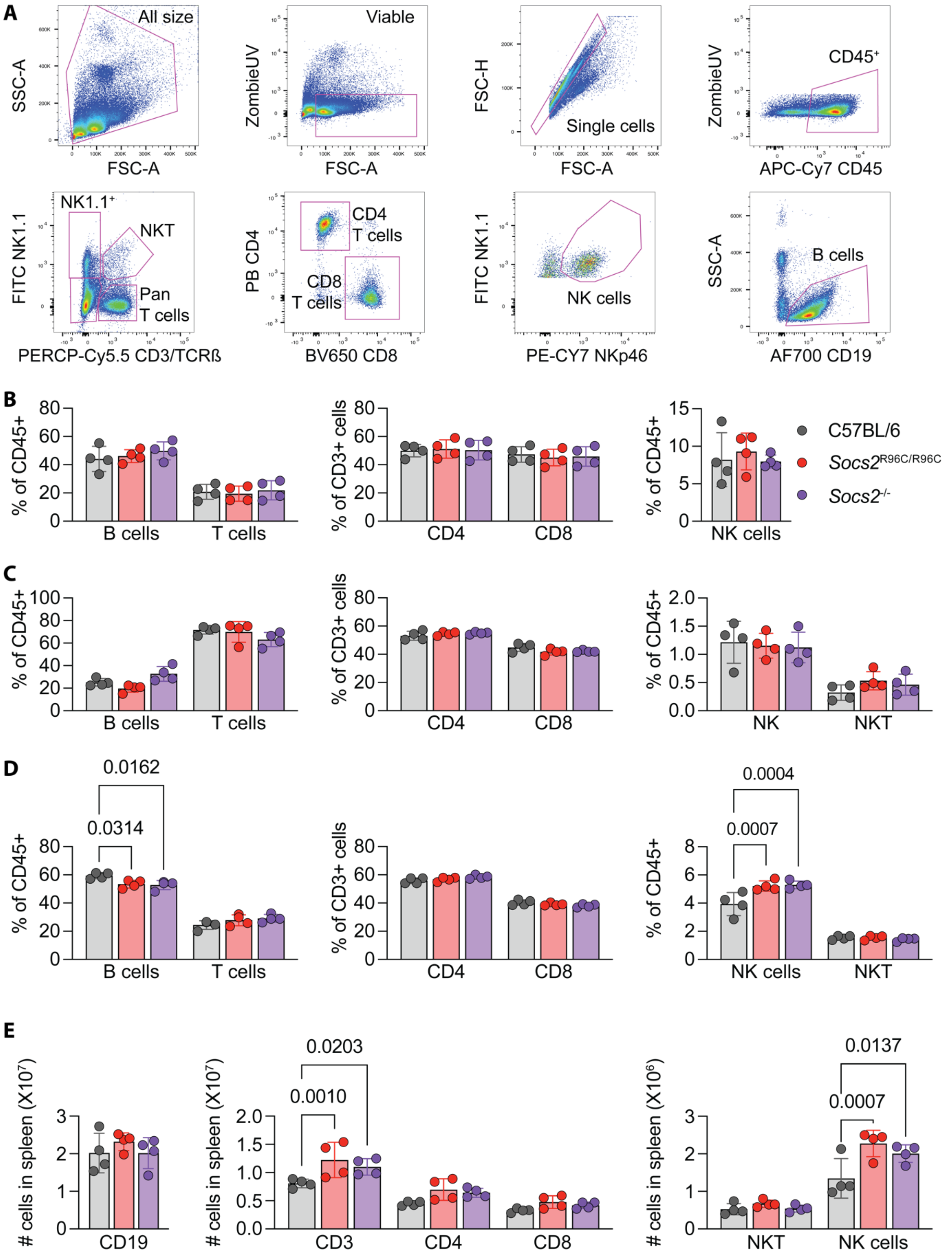

Supplementary Figure 3. Homozygous *Socs2*$^{R96C/R96C}$ and *Socs2*$^{-/-}$ mice display increased numbers of splenic NK cells. Single cell suspension peripheral blood mononuclear cells (PBMCs), splenocytes and lymphocytes from 8-12-week-old control C57BL/6 (black),

Socs2$^{R96C/R96C}$ (red) and *Socs2*$^{-/-}$ (purple) mice were stained with fluorescently conjugated antibodies to various immune markers and analysed by flow cytometry. **(A)** Example gating for the analysis of viable leukocytes (ZombieUV^{-}CD45^{+}), NKT (CD3^{+}TCRß$^{+}$NK1.1^{+}), Pan T cells (CD3^{+}TCRß$^{+}$NK1.1^{-}), CD4 T cells (CD3^{+}TCRß$^{+}$NK1.1^{-}CD4^{+}), CD8 T cells (CD3^{+}TCRß$^{+}$NK1.1^{-}CD8^{+}), Pan B cells (CD3^{-}TCRß$^{-}$NK1.1^{-}CD19^{+}) and NK cells (CD3^{-}TCRß$^{-}$NK1.1^{+}NKp46^{+}). Percentage of various immune populations in the **(B)** blood, **(C)** axial and inguinal lymph nodes, and **(D)** spleen. **(E)** Enumeration of immune populations in the spleen using counting beads (123count eBeads). **(B-E)** Each dot represents cells from an individual mouse. Significance determined by two-way ANOVA with Sidak's multiple comparisons test.

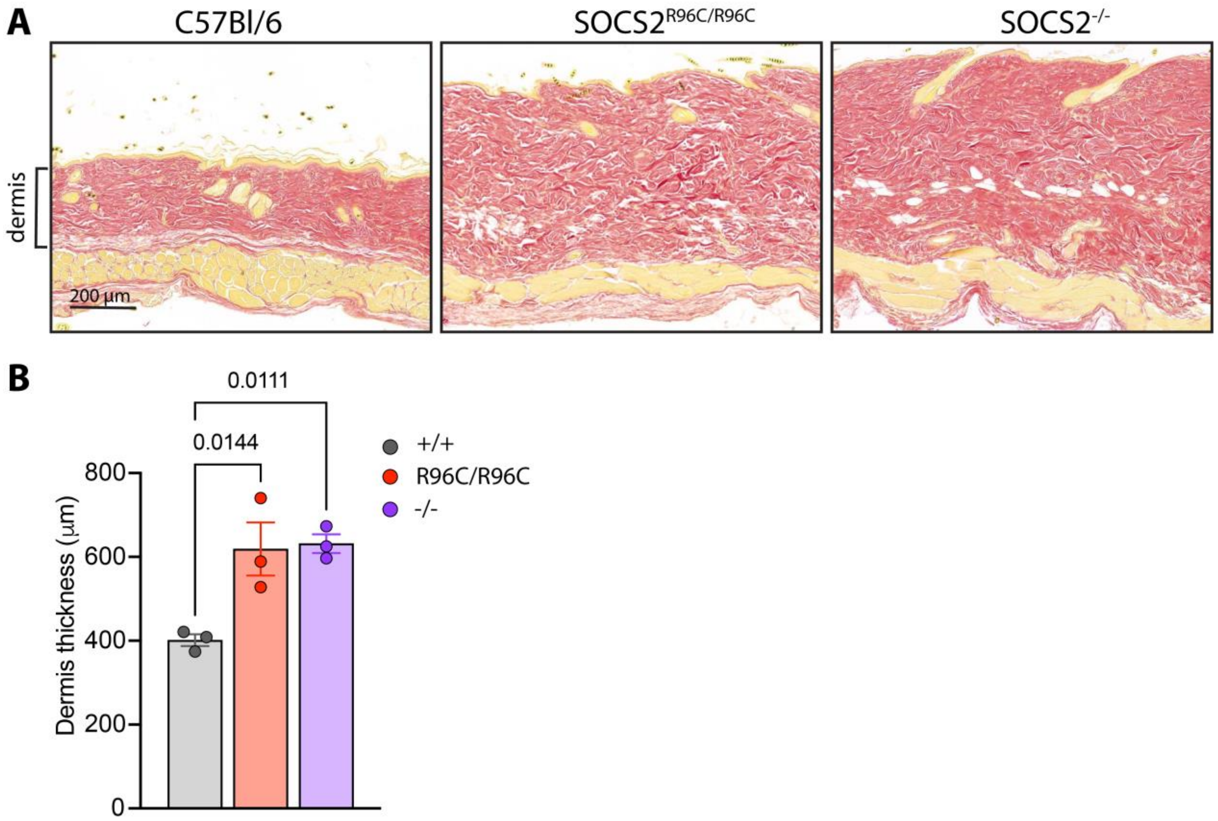

Supplementary Figure 4. Homozygous *Socs2*$^{R96C/R96C}$ and *Socs2*$^{-/-}$ mice display thickening of the skin. (A) Van Giessen-stained dorsal skin section from 10-week-old male WT, *Socs2*$^{R96C/R96C}$ and *Socs2*$^{-/-}$ mice. *Socs2* mutant and null mice show increased collagen deposits and a thickened dermis. Scale bar = 200 µm. Representative images of 3 mice of each genotype. **(B)** Quantification of dermis thickness. n=3 mice/genotype. Data were analyzed using a one-way ANOVA.

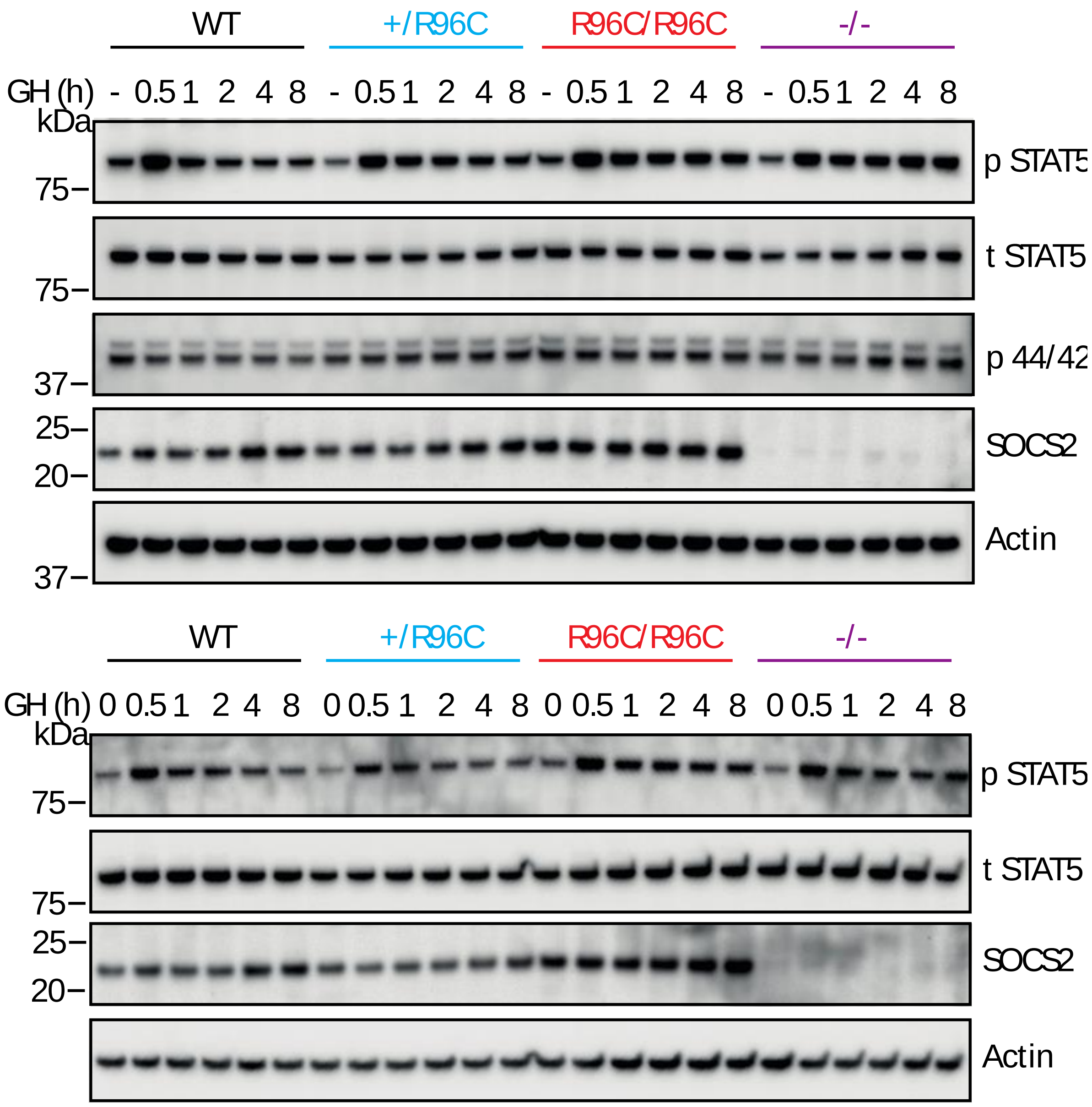

Supplementary Figure 5. *Socs2*$^{R96C/R96C}$ and *Socs2*$^{-/-}$ MEFs display prolonged growth hormone signal activation. *Socs2*$^{+/+}$, *Socs2*$^{R96C/+}$, *Socs2*$^{R96C/R96C}$ and *Socs2*$^{-/-}$ MEFs were treated with 50 ng/mL of GH, lysed and analysed by immunoblotting with antibodies to the indicated proteins. P: phosphorylated, T: total. Two additional independent experiments related to **Figure 4.**

Supplementary Table 1. *Socs2^{R96C}* genotyping primers.

	Allele	Forward Primer (5'-3')	Reverse Primer (5'-3')	PCR Product (bp)
**Standard genotyping**	*Socs2^{R96C}*	AGCTTTCCACTTTGTCCCCTA	ATCTGAATTTCCCATCTTGGTACTCAAT ACAT	766
	Socs2$^{+/+}$	GCTGGACCGACTAACCTGC	AGCATGGTCAGCTTAACGGAA	901
**NGS* genotyping**		GTGACCTATGAACTCAGGAGTCTGAC TGTTAATGAAGCCAAAGAG	CTGAGACTTGCACATCGCAGCGTGAACA GTCCCATTCCGTG	290

*Next generation sequencing
